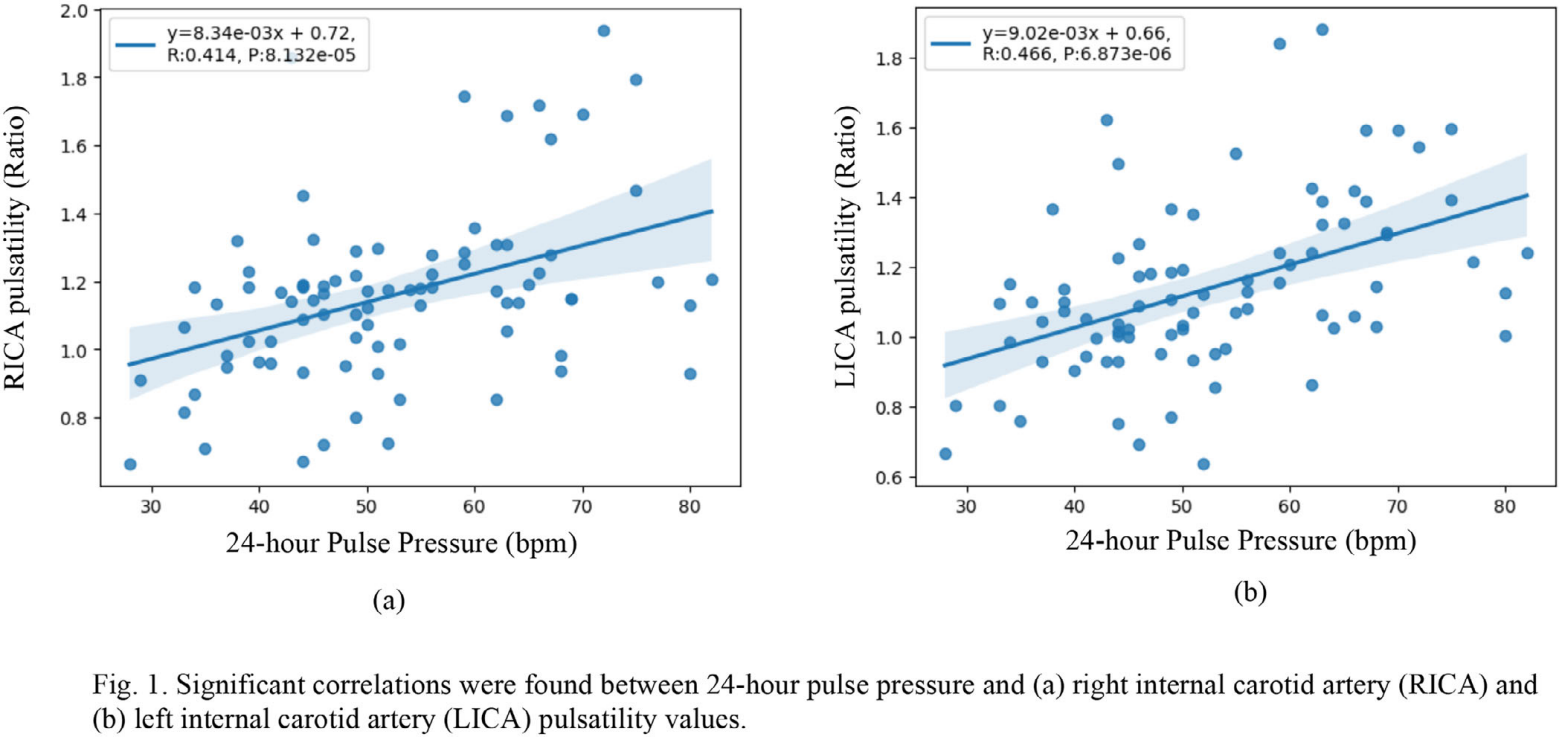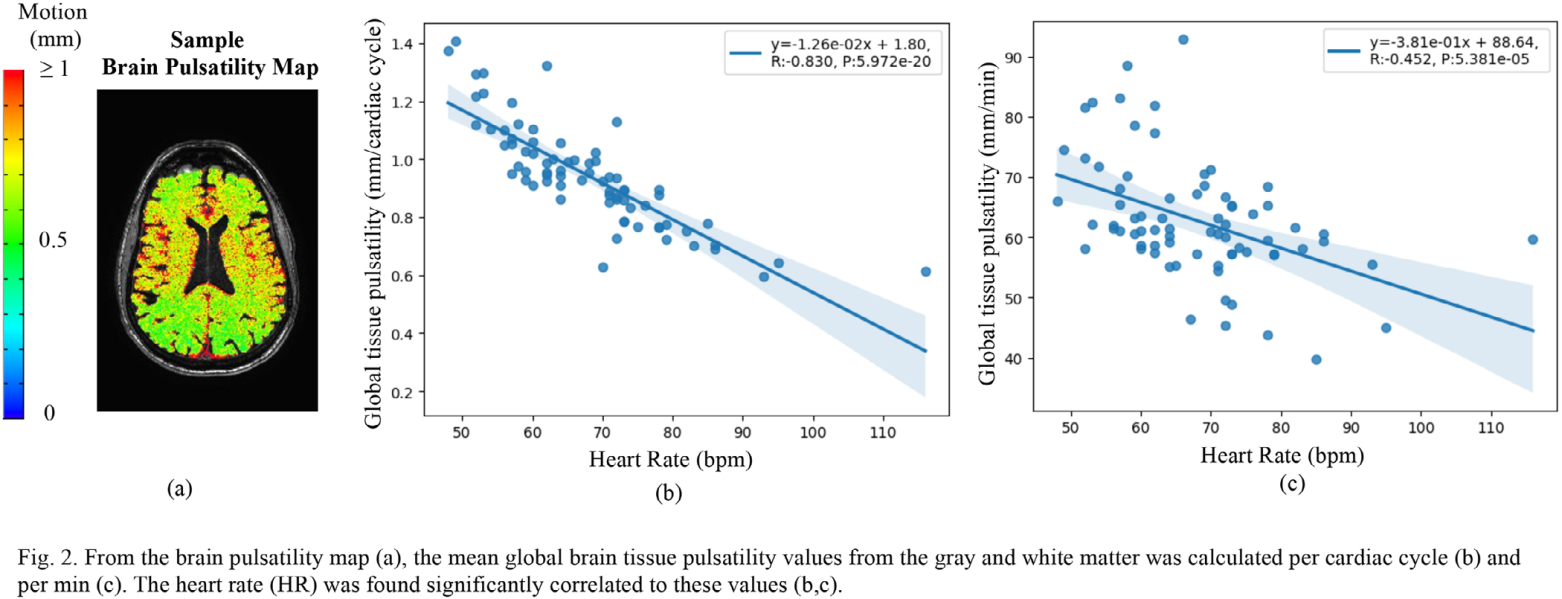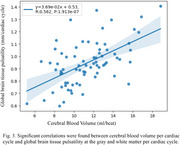# Associations of Blood Pulse Pressure and Heart Rate with Brain Blood Flow and Tissue Pulsatility in Older Adults

**DOI:** 10.1002/alz70856_102253

**Published:** 2025-12-25

**Authors:** David C Zhu, Josh Hubert, Norman Scheel, Ashley Murillo, Danielle Pittman, John Giacona, Brooke Bates, Anna Tomlinson, Wanpen Vongpatanasin, Rong Zhang

**Affiliations:** ^1^ Albert Einstein College of Medicine, Bronx, NY, USA; ^2^ Michigan State University, East Lansing, MI, USA; ^3^ University of Texas Southwestern Medical Center, Dallas, TX, USA; ^4^ Institute for Exercise and Environmental Medicine, Texas Health Presbyterian Hospital Dallas, Dallas, TX, USA

## Abstract

**Background:**

In the HIPAC Trial (Hypertension, Intracranial Pulsatility and Brain Amyloid‐beta Accumulation in Older Adults) (NCT03354143), one aim is to understand the relationships between blood pressure, heart rate, intracranial arterial blood flow pulsatility, and brain tissue pulsatility.

**Methods:**

We obtained high‐quality baseline MRI data from 85 subjects (65 +/‐ 6 years old, 38 male) on a GE Discovery 3T MR750W MRI scanner. 2D CINE Phase Contract (PC) MRI was used to measure brain blood flows and brain tissue pulsatility. We quantified the blood flow pulsatility at the right and left internal carotid arteries (ICA) based on the difference of the maximum and minimal flows, normalized by the mean flow across all 32 time points of the cardiac cycle. Cerebral blood volume per cardiac cycle (CBV‐C) was calculated based on the sum of blood flows from internal carotid and vertebral arteries over one cardiac cycle. To assess brain tissue pulsatility, an axial slice across the lateral ventricle was acquired with VENC = 5 cm/sec, three velocity directions and 16 time points. The brain tissue pulsatility was quantified by the total tissue movement (mm) over one full cardiac cycle, and then was converted to mm per minute. 24‐hour systolic blood pressure (SBP) and diastolic blood pressure (DBP) were collected, and pulse pressure (PP) was calculated.

**Results:**

Significant positive correlations were found between PP and right ICA (*R* = 0.414, *P* = 8.13 x 10^‐5^) and left ICA (*R* = 0.466, *P* = 6.87 x 10^‐6^) blood flow pulsatility (Figure 1); negative correlations were found between heart rate and global brain tissue pulsatility per cardiac cycle (*R* = ‐0.830, *P* = 5.97 x 10^‐20^), as well as per minute (*R* = ‐0.452, *P* = 5.38 x 10^‐5^) (Figure 2). Heart rate was also negatively correlated with CBV‐C (*R* = ‐0.577, *P* = 2.61 x 10^‐9^) while CBV‐C was positively correlated with global brain tissue pulsatility per cardiac cycle (*R* = 0.562, *P* = 1.91 x 10^‐7^) (Figure 3).

**Conclusion:**

In older adults, blood pulse pressure is positively correlated with arterial blood flow pulsatility, and heart rate is negatively correlated with brain tissue pulsatility.